# Eye Movement Patterns as Robust Biomarkers for Schizophrenia Identification Using a Novel Data Transformation Approach

**DOI:** 10.3390/jemr19030051

**Published:** 2026-05-11

**Authors:** Lijin Huang, Senhao Li, Zhi Liu, Dan Zhang, Lihua Xu, Tianhong Zhang, Jijun Wang

**Affiliations:** 1 School of Communication and Information Engineering, Shanghai University, Shanghai 200444, China; 2 Shanghai Key Laboratory of Psychotic Disorders, Shanghai Mental Health Center, Shanghai Jiaotong University School of Medicine, Shanghai 200030, China

**Keywords:** schizophrenia, eye movement abnormalities, sparsity-scoring kernel entropy component analysis, data transformation, machine learning, semantic images

## Abstract

Although eye movement abnormalities are documented in schizophrenia (SZ), their translation into objective diagnostic biomarkers remains limited. In this study, we propose a novel identification framework that integrates a Sparsity-Scoring Kernel Entropy Component Analysis (SSKECA) algorithm with a multidimensional eye movement feature set. A total of 40 patients with SZ and 50 healthy controls (HC) completed a free-viewing task involving 100 distinct semantic images. The proposed SSKECA algorithm optimizes multidimensional feature representations to capture latent eye movement patterns characteristic of SZ. The SSKECA–AdaBoost model achieved competitive performance, with an accuracy of 0.933 and an area under the receiver operating characteristic curve (AUC) of 0.960. Notably, when restricted to only 25 highly discriminative images, the SSKECA–XGBoost model achieved an accuracy of 0.922. Feature ablation analyses not only reproduced previously reported eye movement findings but also highlighted additional atypical patterns. Misclassification analyses revealed more pronounced eye movement deficits in incorrectly classified SZ patients. Overall, the proposed framework translates complex eye movement patterns into robust indicators for subject-level identification, offering a practical and efficient tool to support objective assessment in SZ.

## 1. Introduction

Schizophrenia (SZ) is a heterogeneous psychiatric disorder that arises from the complex interaction of genetic, environmental, and neurobiological factors. It is characterized by cognitive deficits, emotional dysregulation, and behavioral disturbances [1]. Typically emerging in late adolescence or early adulthood, SZ affects approximately 0.3% of the global population and is a leading cause of disability [2]. Despite this significant burden, clinical diagnosis remains challenging. Current diagnostic protocols rely heavily on subjective assessments, such as the MINI-International Neuropsychiatric Interview and the Positive and Negative Syndrome Scale (PANSS), which are based on patient self-reports and clinician observations [3,4]. This reliance on subjectivity can lead to misdiagnosis and delayed treatment. Consequently, there is a critical need to develop objective, quantifiable biomarkers to enable earlier identification.

Eye-tracking technology provides a robust, non-invasive method for quantifying visual processing and eye movement control [5]. Research across diverse paradigms has consistently documented distinct eye movement abnormalities in individuals with SZ relative to healthy controls (HC). These deficits include impaired sensorimotor integration, evidenced by reduced velocity and accuracy in smooth and predictive pursuit tasks [6,7,8]. Similarly, in exploratory eye movement tasks, individuals with SZ demonstrate restricted visual scanning and lower amplitude, which are linked to top-down attentional deficits [9,10]. Furthermore, fixation stability assessments reveal decreased visual engagement, characterized by variable durations and excessive saccades away from target regions [11,12]. Recently, video-based paradigms have been introduced to further extend free-viewing eye-tracking to dynamic visual stimuli [13]. Of particular relevance to this study, free-viewing paradigms mimicking naturalistic vision indicate that individuals with SZ exhibit narrower scanning ranges, reduced saccadic velocity, and altered pupil dynamics [6,14]. However, while these group-level differences confirm the diagnostic potential of eye-tracking metrics, existing analyses have relied predominantly on traditional statistical methods. Such methods often fail to capture complex structural patterns within the data, highlighting a critical methodological gap that this study aims to address.

In response, recent studies have increasingly focused on integrating multi-dimensional eye movement features with machine learning (ML) techniques to identify more sensitive and specific eye movement biomarkers for SZ. Huang et al. [15] incorporated visual salience mechanisms to construct richer, multi-structural feature representations, achieving an accuracy of 0.871 and a sensitivity of 0.900 in distinguishing SZ from HC. Iwauchi et al. [16] integrated facial expression-specific eye movement patterns into a weighted convolutional neural network (CNN) framework, resulting in a classification accuracy of 0.645 and a sensitivity of 0.667. Beyond binary classification, further efforts have sought to address disease heterogeneity and progression. Chen et al. [17] identified discriminative eye movement biomarkers capable of differentiating among clinical high-risk (CHR), first-episode SZ (FSZ), and HC groups, achieving accuracies of 0.800 (FSZ vs. HC), 0.813 (FSZ vs. CHR), and 0.860 (CHR vs. HC) based on support vector machine–recursive feature elimination (SVM–RFE). From a methodological perspective, Song et al. [18] proposed MSNet, a mean-shift-based network designed to enhance stimulus-aware feature extraction, which achieved an accuracy of 0.831 and a specificity of 0.871.

Despite these advancements, two fundamental methodological limitations persist, hindering the reliable application of these models to individual-level diagnoses. The first limitation involves suboptimal feature transformation. Conventional transformation methods, such as principal component analysis (PCA), kernel PCA (KPCA), and kernel entropy component analysis (KECA), prioritize variance maximization or kernel-induced mapping. Consequently, these methods often fail to detect disorder-specific pattern irregularities in eye movement data [19]. The second limitation is restricted feature representation. Most studies rely on a limited set of feature types, failing to capture the multidimensional nature of neurocognitive dysfunction in SZ and the stimulus-dependent specificity of visual processing deficits.

This study systematically evaluated whether the novel Sparsity-Scoring Kernel Entropy Component Analysis (SSKECA) algorithm outperforms traditional approaches in identifying SZ. Using a multidimensional eye movement feature set optimized by SSKECA, we aimed to isolate the most discriminative eye movement biomarkers. Furthermore, through the integration of diverse classifiers, we elucidated data-driven visual processing patterns during free viewing, specifically identifying patient sensitivity variations across different semantic image categories. These insights enabled the development of a streamlined, SZ-specific stimulus set, offering a robust foundation for advancing clinical diagnostic tools and facilitating their practical application.

## 2. Materials and Methods

### 2.1. Participants

We expanded the public dataset originally reported by Huang et al. [15], which was initially collected to investigate eye movement abnormalities in SZ during a naturalistic image-viewing task. This original dataset included 40 patients with SZ and 30 HC. To address the notable age difference observed between the two groups, an additional 20 HC were recruited. Consequently, the refined dataset used in this study consisted of 40 individuals with SZ and 50 HC. The gender distribution remained balanced between groups. The slightly higher age and fewer years of education in the SZ group are inherent clinical features resulting from the illness’s interference with academic progression. Therefore, the cohorts were considered appropriately matched for this study.

Inclusion criteria for all participants were: (a) age between 18 and 60 years; (b) absence of intellectual disability, neurological disorders, substance dependence, or color blindness; (c) absence of high myopia; and (d) right-handedness. Exclusion criteria included a history of head trauma, electroconvulsive therapy, or severe medical conditions that could affect eye movement. All participants were fully informed of the study’s purpose, procedures, and potential risks, and written informed consent was obtained prior to the assessment. The study was approved by the Ethics Committee of Shanghai Mental Health Center (SMHC). Detailed demographic and clinical characteristics are summarized in Table 1.

### 2.2. Stimulus Database

The visual stimulus database used in this study was adopted from [15]. It comprises 100 images with rich semantic content, selected to approximate ecologically valid free-viewing conditions. These images are organized into 20 distinct categories, with the number of images per category ranging from 2 to 10. The distribution of images across categories is summarized in Supplementary Table S1.

### 2.3. Free-Viewing Task

Eye movement data were collected in a dedicated testing room using an EyeLink 2000 desktop eye tracker (SR Research, Ottawa, ON, Canada).The system was paired with a 17-inch LCD display with a resolution of 800 × 600 pixels. Participants were seated at a fixed viewing distance of approximately 60 cm. A sampling rate of 500 Hz was selected to capture fine-grained gaze dynamics. To ensure data quality for participants wearing corrective lenses, pupil-only tracking was employed to minimize artifacts from reflections on eyeglasses [20]. Prior to data collection, a standard nine-point calibration procedure was performed.

The free-viewing task was implemented using Experiment Builder software (SR Research, Version 2.3.1) to ensure precise timing control. The experimental sequence proceeded as follows: (a) a central fixation cross (20 × 20 pixels) was displayed on a white background for 1.5 s to establish initial gaze alignment; (b) 100 images from the stimulus database were presented in a randomized order, with each image displayed for 5 s, yielding 100 experimental trials per participant; and (c) participants were instructed to “view the images naturally, as in daily life”, with no explicit task requirements (e.g., memorization or evaluation). The total session duration was approximately 10–12 min, with a 1-min break provided midway to mitigate fatigue. This paradigm was designed to capture eye movement patterns reflecting real-world cognitive functioning, distinct from structured tasks such as smooth pursuit. Eye movement data (including fixations, saccades, and pupil dynamics) were recorded continuously and stored in EyeLink Data File (EDF) format for subsequent preprocessing.

A systematic data cleaning procedure was employed to exclude invalid data according to the following criteria: (1) fixations and saccades occurring entirely outside the display area were removed; (2) saccades with amplitudes <1° or frequencies between 1 and 2 Hz were identified as involuntary microsaccades and subsequently excluded; and (3) fixations with durations shorter than 80 ms or longer than 2000 ms were discarded [21,22].

### 2.4. Hybrid Feature Set

**Figure 1:**
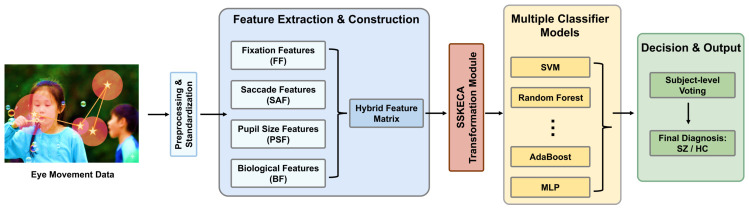
Overall pipeline for SZ identification. Eye movement data are preprocessed and standardized, and fixation, saccade, pupil, and biological features are extracted to construct a hybrid feature matrix. The matrix is then transformed by SSKECA and evaluated using multiple classifiers, followed by subject-level voting to generate the final classification (SZ/HC). The fixation point is denoted by a star symbol ⋆.

Eye movement abnormalities in SZ reflect complex neurocognitive impairments across multiple domains. Therefore, a single type of eye movement feature is insufficient to fully capture the distinguishing characteristics between SZ and HC groups [15,23]. To address this, we constructed a hybrid feature matrix to comprehensively characterize the abnormal visual exploration behavior observed in SZ. The overview of the proposed SSKECA–ML framework is shown in Figure 1.

**Fixation features (FF):** Fixations represent periods of relative stability where gaze is maintained on a target, constituting a critical phase for visual information processing. In individuals with SZ, this process is disrupted due to hypoactivation in the frontoparietal network [24,25]. In this study, we extracted a total of nine fixation features. Among these, we adopted two specific hand-crafted features from [15]: fixation skewness (FS) and outside fixation count (OFC). FS quantifies the central bias of the fixation distribution, while OFC measures the number of fixations falling outside the image borders during a single sample.

**Saccade features (SAF):** SAF serve as valuable markers for cognitive analysis, offering insights into underlying cognitive processes [26]. Defined as rapid shifts of gaze between fixation points, saccades are characterized by parameters such as amplitude, velocity, and duration. These parameters are largely influenced by environmental context and target location [27]. We extracted four SAF from the original eye movement data.

**Pupil size features (PSF):** PSF capture real-time physiological and spatial dynamics of eye movements, providing direct insights into autonomic and attentional responses. These metrics are particularly valuable as indices of autonomic nervous system activity, which is known to be dysregulated in SZ. In this study, we extracted a total of seven PSF from the eye movement data. Specifically, we adopted two hand-crafted features from Huang et al. [15]: the dynamic range of pupil size (DR) and the pupil size ratio (SR).

**Biological features (BF):** BF are derived metrics that integrate FF and SAF to reflect broader visual exploration patterns, linking eye movement behavior to clinical phenotypes [28]. In this study, we extracted four BFs. It is important to note that “duration” in this context refers to the total time of a single sample (from the start to the end of data collection). Specifically, we employed valid viewing duration (VVD) [15] to quantify the subjects’ interest in the image. VVD is defined as the cumulative duration of all fixations within a sample.

To summarize, a complete 24-dimensional feature matrix was constructed and validated. Statistical analysis was performed using Welch’s two-sample *t*-test for each feature to assess inter-group differences. In addition, within-group means and standard deviations were calculated to describe feature distributions, and Cohen’s *d* was calculated to quantify the standardized effect size of group differences. A comprehensive summary of the feature matrix and statistical results is presented in Table 2.

### 2.5. Innovative Data Transformation Framework

Raw and multi-dimensional features present a heightened risk of overfitting [29], and direct analysis of such untransformed data often fails to uncover latent discriminative patterns that are essential for SZ classification [30]. To mitigate these issues, we propose a novel framework termed SSKECA, which integrates adaptive parameter estimation to optimize feature representation and enhance class separability. For comparative evaluation, traditional data transformation methods, including KECA, PCA, and KPCA, were adopted as benchmark approaches.

**Figure 2:**
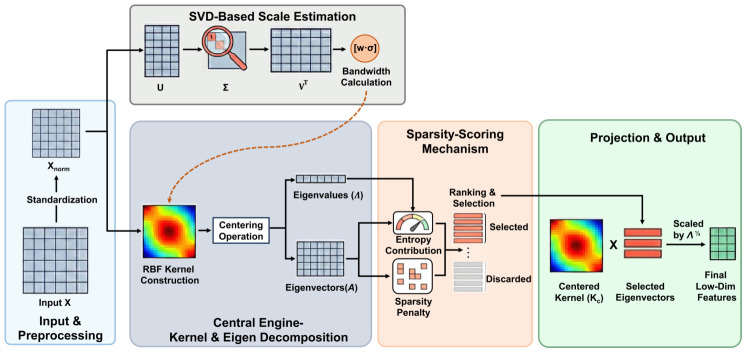
SSKECA transformation module (SSKECA: Sparsity-Scoring Kernel Entropy Component Analysis). The standardized feature matrix is mapped into an RBF-kernel space, where the kernel matrix is centered and decomposed. Informative components are selected using a Sparsity-Scoring scheme, and the selected components are used to produce low-dimensional features for downstream classification. The colors in the kernel maps are used for schematic illustration only and do not represent real-valued heatmaps.

Compared to the original KECA, the proposed SSKECA introduces task-specific enhancements designed for SZ identification. SSKECA ranks components based on entropy contribution, incorporating global response to capture information uncertainty. It employs an $\ell_{1}$-norm constrained sparse selection mechanism that integrates a sparsity penalty into the ranking process, followed by Lasso post-processing to minimize redundancy and overfitting. To ensure numerical stability, the framework applies matrix symmetrization, eigenvalue filtering, and projection offsets, effectively addressing singularity issues inherent in eye movement data. These improvements enable SSKECA to mitigate challenges such as high noise and feature redundancy, thereby enhancing both classification performance and clinical interpretability. The detailed mathematical formulation is presented in the following sequential steps, and the SSKECA transformation is illustrated in Figure 2.

- **Step 1: Data preprocessing**

The input data is defined by a raw hybrid feature matrix $X\in R^{N\times D}$, where *N* denotes the number of samples and *D* denotes the feature dimension. To eliminate scale bias between features, Z-score standardization was applied to each feature. The standardized feature matrix $X_{\mathrm{norm}}$ is defined as:

(1) $$X_{\mathrm{norm},i,j}=\frac{X_{i,j}-\mu_{j}}{\sigma_{j}},$$

where $\mu_{j}$ and $\sigma_{j}$ represent the mean and standard deviation of the *j*-th feature, respectively.

- **Step 2: Adaptive parameter estimation**
  Kernel Density Estimation (KDE) and kernel-based learning algorithms are widely used in biomedical data analysis, where kernel bandwidth directly determines model performance [31]. The bandwidth parameter involves a critical trade-off: a narrow bandwidth risks overfitting to noise, while a wide bandwidth risks underfitting and losing important structural information. To mitigate this challenge, our proposed SSKECA framework incorporates an adaptive bandwidth estimation mechanism, which leverages Singular Value Decomposition (SVD) to derive bandwidth values from the intrinsic structural characteristics of the data matrix, rather than relying on arbitrary fixed values.
  1. *SVD-based structural scale estimation.* Perform SVD on the standardized matrix:
    (2) $$X_{\mathrm{norm}}=U\Sigma V^{⊤},$$
    where U and $V^{⊤}$ are the left and right singular vector matrices, respectively, and $\Sigma =\mathrm{diag}(\sigma_{1},\sigma_{2},\ldots ,\sigma_{D})$ is the diagonal matrix of singular values, with $\sigma_{1}$ denoting the largest singular value.
  2. *Bandwidth computation.* Based on the SVD results, the data-adaptive bandwidth parameter BW is calculated to control the local action range of the RBF kernel:
    (3) $$\mathrm{BW}=w\cdot \frac{\sigma_{1}}{\sqrt{D}},$$
    where *w* is a tunable scaling factor used to fine-tune the bandwidth according to the distribution characteristics of eye movement data, and *D* represents the feature dimension.

- **Step 3: Kernel matrix construction**

The radial basis function (RBF) kernel function for two normalized samples $x_{i},x_{j}\in X_{\mathrm{norm}}$ is defined as

(4) $$K_{i,j}=\exp\;\left(-\frac{{∥x_{i}-x_{j}∥}^{2}}{2\;{\mathrm{BW}}^{2}}\right),$$

where ${∥x_{i}-x_{j}∥}^{2}$ denotes the squared Euclidean distance between $x_{i}$ and $x_{j}$. The kernel matrix $K\in R^{N\times N}$ captures pairwise non-linear similarities between all samples.

- **Step 4: Kernel matrix centering**

To eliminate sample mean bias and ensure unbiased feature decomposition, kernel matrix centering is performed, which is a critical step for the robust extraction of discriminative components. The centered kernel matrix is computed as:

(5) $$K_{c}=HKH,\;H=I_{N}-\frac{1}{N}1_{N}1_{N}^{⊤}.$$

where $I_{N}$ is the N×N identity matrix, and $1_{N}$ is an N×1 vector of ones. This operation centers the kernel matrix around the global sample mean, consistent with conventional statistical assumptions.

- **Step 5: Stable eigendecomposition**

The centered kernel matrix is $K_{c}$. Since $K_{c}$ is symmetric and positive semi-definite, an efficient symmetric eigenvalue decomposition method was used to improve stability and computational speed. The decomposition of $K_{c}$ is expressed as:

(6) $$K_{c}=A\Lambda A^{⊤},$$

where $\Lambda =\mathrm{diag}(\lambda_{1},\lambda_{2},\ldots ,\lambda_{N})$ is a diagonal matrix of eigenvalues sorted in descending order, and $A=[\alpha_{1},\alpha_{2},\ldots ,\alpha_{N}]$ is the corresponding eigenvector matrix, with each $\alpha_{i}\in R^{N}$ denoting the eigenvector associated with $\lambda_{i}$.

- **Step 6: Sparsity-Scoring mechanism**
  The core innovation of SSKECA is implemented through the following sub-steps.
  1. *Eigenvector normalization.* Each eigenvector is normalized to unit $\ell_{2}$ norm:
    (7) $$e_{i}=\frac{\alpha_{i}}{{∥\alpha_{i}∥}_{2}},\;i=1,\ldots ,N.$$
  2. *Entropy contribution.* The entropy contribution of each component is defined as
    (8) $$G_{i}=\sqrt{\lambda_{i}}\cdot \left|e_{i}^{⊤}1_{N}\right|.$$
  3. *Sparsity-Scoring.* A sparsity-score is computed to weight each component by its entropy contribution while penalizing redundant or dense representations, thereby retaining informative components with compact structure.
    (9) $$s_{i}=G_{i}-\beta \cdot {∥\alpha_{i}∥}_{1},$$
    where β controls the sparsity penalty.
  4. *Component ranking and selection.* The top $n_{\mathrm{comp}}$ components are selected by ranking the sparsity scores in descending order. Let I denote the index set of the selected components, defined as
    (10) $$I=\mathrm{TopK}\left({\left\{s_{i}\right\}}_{i=1}^{N},\;n_{\mathrm{comp}}\right),$$
    where $\mathrm{TopK}(\cdot ,n_{\mathrm{comp}})$ returns the indices of the $n_{\mathrm{comp}}$ largest values in the set ${\left\{s_{i}\right\}}_{i=1}^{N}$.

Accordingly, the corresponding eigenpairs are retained as

(11) $$\Lambda_{\mathrm{sel}}=\Lambda_{II},\;A_{\mathrm{sel}}=A_{:I}.$$

where $\Lambda_{\mathrm{sel}}\in R^{n_{\mathrm{comp}}\times n_{\mathrm{comp}}}$ and $A_{\mathrm{sel}}\in R^{N\times n_{\mathrm{comp}}}$ denote the selected eigenvalue and eigenvector matrices, respectively. $\Lambda_{II}$ is the submatrix of Λ obtained by selecting the diagonal entries indexed by I, and $A_{:I}$ is the submatrix of A retaining all rows and the columns indexed by I.

- **Step 7: Feature projection**

The final optimized feature matrix is obtained by projecting the centered kernel matrix onto the selected components:

(12) $$X_{\mathrm{trans}}=K_{c}\cdot A_{\mathrm{sel}}\cdot \Lambda_{\mathrm{sel}}^{-\frac{1}{2}}.$$

### 2.6. Classification and Performance Assessment

A stratified randomization protocol was employed to partition the 90 participants (40 SZ and 50 HC). Crucially, this approach ensured that all data samples from any single participant were assigned exclusively to either the training set or the test set. This precaution prevents data leakage and ensures independent validation. The original 4:5 ratio of SZ to HC was preserved across all cross-validation (CV) folds. Before dividing the folds, subject IDs were randomly permuted to mitigate potential selection bias related to recruitment order or assessment timing. In each CV iteration, four folds formed the training set (n = 72), while the remaining fold served as the independent test set (n = 18), consistently maintaining the 4:5 ratio in both partitions to reflect the original cohort distribution.

KECA, PCA, and KPCA were implemented using standard parameter configurations to benchmark the performance of the proposed SSKECA method. PCA was performed using SVD on the centered feature matrix, consistent with established methodologies in SZ-related ML research [32]. The retained dimensionality was selected based on downstream classification performance rather than a predefined cumulative variance threshold. KECA and KPCA employed the same RBF kernel, with a fixed bandwidth parameter (BW = 3.948) to match the adaptive bandwidth used in SSKECA.

To validate the efficacy of the SSKECA-transformed feature matrix for SZ identification, a range of ML classifiers were selected: Adaptive Boosting (AdaBoost), Random Forest (RF), K-Nearest Neighbors (KNN), Support Vector Machine (SVM), Multi-Layer Perceptron (MLP), EXtreme Gradient Boosting (XGBoost), Light Gradient Boosting Machine (LightGBM), and Convolutional Neural Network (CNN). These classifiers cover ensemble, distance-based, kernel-based, and neural network paradigms, ensuring a comprehensive evaluation of feature adaptability across different classifiers [33]. The 95% confidence intervals (CIs) were computed using 1000 iterations of bias-corrected and accelerated bootstrap resampling (BCa bootstrap) based on subject-level performance metrics. Hyperparameter configurations for the employed ML classifiers are summarized in Table S2 provided in the Supplementary Materials. To further enhance the clinical applicability of the classification framework, a voting algorithm was integrated to determine subject-level diagnostic labels. Specifically, using a threshold criterion of 50%, participants for whom the proportion of sample-level labels exceeded this value were designated as SZ, while others were classified as HC. This approach reduces the impact of individual sample noise on diagnostic results, consistent with real-world clinical assessment. All models were trained under the unified CV and data partitioning protocols described above, with consistent experimental settings to ensure fair comparison and reproducibility.

For comparison, we additionally included three representative methods previously used for eye-movement-based disorder recognition, including GPI–LSTM and GPI–GRU from Zhou et al. [34] and ABG-LSTM from Li et al. [35]. GPI–LSTM and GPI–GRU are sequence-based methods that can effectively capture temporal dependencies in eye-movement sequences. ABG–LSTM is an appearance-based framework that first estimates gaze from two-eye images and then uses the derived gaze information for classification. The relevant results are shown in Section 3.1.

### 2.7. Biomarker Research

In the study of eye movement biomarkers for SZ, feature ablation experiments restricted to a single analytical perspective are insufficient to capture the inherent heterogeneity and complexity of the disorder. Accordingly, we propose a dual-level feature ablation strategy grounded in SSKECA-transformed representations, which jointly incorporates both sample-level and subject-level analyses. Both baseline and ablation conditions were evaluated using SSKECA-based transformation (BW = 3.948), followed by AdaBoost classification. Within this framework, feature importance was quantified as the reduction in classification accuracy (∆ACC) induced by ablating each individual feature relative to the full-model baseline.

Sample-level ablation: From a population research perspective, sample-level ablation is essential. Since SZ is a highly heterogeneous neuropsychiatric disorder, its core pathological mechanisms often lead to shared, population-level traits [36]. By systematically removing entire categories of eye movement features across all participants, this method identifies attributes with consistent discriminative power across the entire sample cohort. Specifically, we first established a performance baseline using the complete feature set processed by SSKECA. We then iteratively removed individual features one by one, retraining the classification model on the remaining features after SSKECA transformation. By comparing the accuracy against the baseline, we evaluated the specific contribution of each feature to the model’s overall discriminative power. A decline in performance indicates that the removed feature serves as a potential biomarker with population-level stability for SZ identification.

Subject-level ablation: In contrast, subject-level ablation addresses the clinical challenges imposed by data noise and individual variability. By simulating incomplete data scenarios through iterative feature masking, this method evaluates model robustness against real-world artifacts. Crucially, it uncovers subject-specific patterns often obscured by group-level metrics, thereby facilitating personalized precision assessment. Methodologically, we enforced a strict subject-wise data partition, ensuring that all samples from a given participant were allocated exclusively to either the training or testing set to prevent data leakage. We then iteratively masked individual features for each subject and processed the remaining data via SSKECA. Ultimately, the diagnostic outcome for each subject was determined by aggregating sample-level predictions through a majority voting mechanism. The specific results are presented in Section 3.2.

### 2.8. Semantic Analyses

The free-viewing image database consists of 20 categories, each containing implicit semantic information [15]. Although visual cognitive impairments in SZ are recognized, the specific image categories that trigger distinct responses remain unclear. Furthermore, techniques to accurately capture these associations are currently lacking. To address this, we used semantic analysis based on SSKECA to systematically quantify differences in patients’ responses to various image categories, establishing a quantitative link between disease state and semantic preferences. For each image category, classification accuracy was calculated by employing SSKECA–AdaBoost, defined as the ratio of correctly classified eye movement samples to the total samples within that category. Based on prior findings from eye-tracking studies of SZ [15] and the practical need to reduce the number of images, thereby shortening data acquisition time, we adopted an accuracy threshold of 0.85 as a criterion for selecting images with relatively high discriminative potential. Based on these identified categories, we constructed a targeted image database for SZ. This dataset was then processed using SSKECA and analyzed using the employed classifiers. Individual performance was evaluated using Accuracy, Precision, Recall, F1-score, and area under the receiver operating characteristic curve (AUC). Detailed results can be found in Section 3.3.

## 3. Results

### 3.1. Model Performance

Prior to data transformation, the baseline classification accuracy achieved by AdaBoost was 0.900 ± 0.082. To evaluate the overall effectiveness of different data transformation algorithms, we compared the performance of each algorithm when paired with its best-performing classifier (Table 3). Among all methods, the proposed SSKECA algorithm combined with the AdaBoost classifier demonstrated competitive performance. Specifically, it achieved a classification accuracy of 0.933, while reducing the feature dimensionality from 24 to 21. These results indicate that SSKECA achieves an optimal balance between information compression and the preservation of discriminative information. In contrast, the traditional linear method PCA, although reducing the feature dimensionality to 14, yielded lower accuracy (0.911) than SSKECA. Compared with its entropy-based predecessor, SSKECA exhibited a marginal improvement over KECA (Accuracy = 0.922). Notably, although KPCA also achieved substantial dimensionality reduction, its classification accuracy was 0.022 lower than that of SSKECA. This performance gap highlights the advantage of SSKECA’s adaptive bandwidth mechanism in extracting more discriminative feature representations. These results suggest that SSKECA is a suitable feature dimensionality reduction method for the task considered in this study.

To further examine the generalizability and robustness of the features learned by SSKECA, we evaluated its performance across the different classifiers, as summarized in Table 4. The results indicate that the feature representations produced by SSKECA are not dependent on any specific classification model and consistently maintain strong and stable performance across all classifiers. Specifically, the SSKECA–AdaBoost combination achieved the best overall performance. In combination with MLP, it achieved the highest AUC of 0.970. When paired with SVM and XGBoost, SSKECA also attained a high accuracy of 0.911. Notably, even when coupled with the relatively simple KNN classifier, the model performance remained at a respectable level (Accuracy = 0.878). More importantly, all classifier combinations yielded an accuracy of no less than 0.878. The 95% BCa CIs revealed that AdaBoost, MLP, SVM, and XGBoost yielded relatively compact intervals for several metrics, suggesting consistent performance across CV folds. These results collectively demonstrate that the features obtained after SSKECA reduction exhibit favorable discriminative capability and robust generalizability.

To further evaluate the proposed framework, we compared SSKECA–AdaBoost with three representative methods, as summarized in Table 5. The results showed that SSKECA–AdaBoost consistently achieved higher values across all evaluation metrics than the compared methods. Specifically, it attained an accuracy of 0.933, exceeding those of GPI–LSTM (0.711), GPI–GRU (0.767), and ABG–LSTM (0.756). Similar trends were observed for precision, recall, F1 score, and AUC. In particular, SSKECA–AdaBoost achieved an AUC of 0.960, whereas the AUC values of the three comparison methods ranged from 0.770 to 0.808. These findings further support the effectiveness of the proposed method for SZ identification.

The complete performance results for all combinations of data transformation algorithms and classifiers are provided in Supplementary Table S3.

### 3.2. Biomarker Research Results

**Figure 3:**
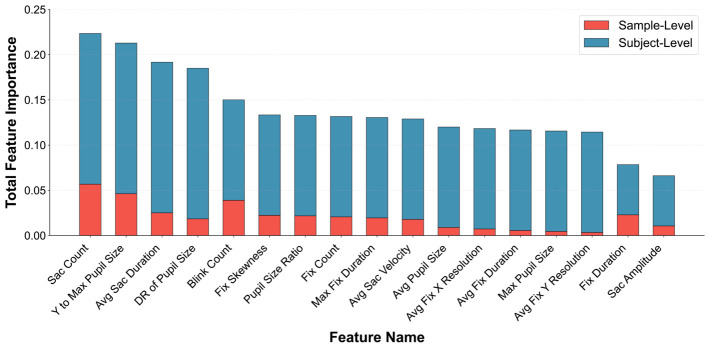
Stacked feature importance of sample- and subject-level eye movement metrics.

To systematically quantify the contribution of individual eye movement biomarkers to diagnostic performance, feature importance was evaluated using an ablation-based accuracy drop method across two complementary dimensions. See Section 2.7 for detailed steps. As shown in Figure 3, substantial heterogeneity in feature importance was observed across different eye movement metrics. Only features exhibiting positive importance were retained, resulting in a total of 17 valid features. The overall importance of these features displayed a clear hierarchical structure. Specifically, the top four features (Sac Count, Y to Max Pupil Size, Avg Sac Duration, and DR of Pupil Size) showed markedly higher total importance values (exceeding 0.22, 0.21, 0.19, and 0.18, respectively) compared with mid-tier features (ranked 5–11, approximately 0.12–0.15) and lower-tier features (the remaining six features, approximately 0.07–0.11). Among them, Sac Count exhibited the highest total importance, indicating that it is the most influential metric for diagnostic performance.

For the majority of features, subject-level importance constituted a larger proportion of the total importance, indicating that inter-individual differences in eye movement patterns play a more prominent role in the model’s decision-making process than intra-individual variations captured at the sample level. In contrast, a small subset of features (e.g., Fix Duration) exhibited relatively stronger sample-level contributions, underscoring their specific utility in capturing fine-grained intra-individual eye movement characteristics.

A density scatter plot illustrating the relationship between sample-level and subject-level feature importance is shown in Supplementary Figure S1. Point density was color-coded using KDE, and a global linear trend corresponding to a Pearson correlation coefficient (r = 0.502) was overlaid. The plot suggests a moderate positive association between the two importance dimensions, with most features clustering in a region of moderate importance across both levels. In contrast, a few features located in low-density regions display divergent patterns, reflecting heterogeneity in their relative contributions.

### 3.3. Semantic Analyses Results

**Figure 4:**
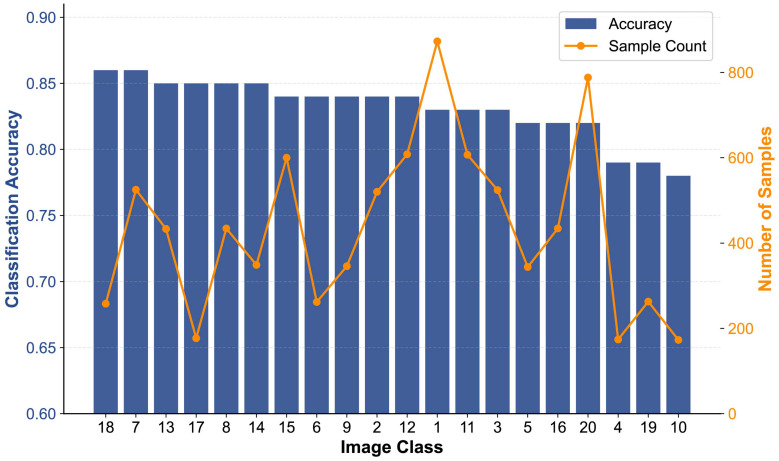
Classification accuracy and sample count across image classes. Image class indices correspond to category labels and stimulus counts listed in Supplementary Table S1. Sample count denotes the total number of eye movement samples aggregated across subjects.

The number of stimulus images and the corresponding category labels are summarized in Supplementary Table S1. The classification outcomes of eye movement samples within each category were aggregated to compute the average classification accuracy, reflecting how effectively each category distinguishes SZ from HC. Figure 4 illustrates the classification accuracy and sample count across 20 image categories. Classification accuracy remains robust (0.82–0.86) for most categories, with peak performance (0.86) observed in structured visual types (e.g., Indoor scenes, Class 7). In contrast, the lowest accuracy (0.78–0.79) occurs in more abstract or less-structured categories, such as Line drawings (Class 10), Sketches (Class 19), and Black-white images (Class 4). Sample count varies substantially across categories, ranging from approximately 170 to 880 samples, and peaks at nearly 880 for Action, which also maintains a relatively high accuracy (0.83). Collectively, these results indicate that classification performance depends more strongly on category-specific visual characteristics (e.g., structural organization and content) than on sample size, with structured image categories supporting more reliable discrimination between groups.

**Figure 5:**
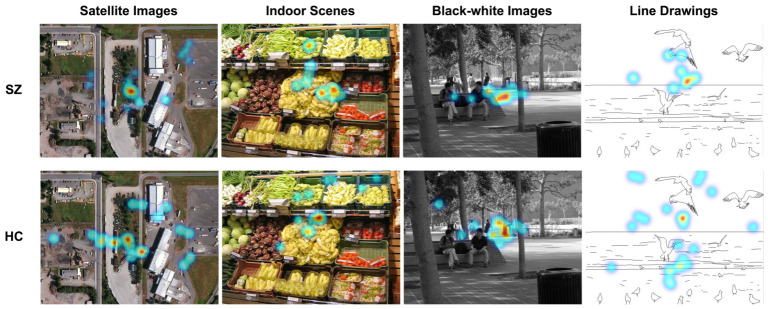
Fixation density maps of one subject in the SZ group or the HC group. Satellite images and indoor scenes represent examples of highly discriminative images, whereas black-white images and line drawings correspond to least discriminative cases.

To facilitate visual comparison, Figure 5 presents representative images with the highest and lowest discriminative power, overlaid with fixation density maps from the control and patient groups. Overall, clear visual differences between the two groups are observed for images with high classification accuracy (most discriminative), whereas fixation patterns appear largely similar for images with low classification accuracy (least discriminative). In highly discriminative images, the HC group distributes attention across multiple semantic regions, including areas distant from the image center (e.g., clustered vehicles in satellite images and vegetable stalls in indoor scenes), while the SZ group tends to focus on a much narrower range, often limited to a single object in satellite images, consistent with the restricted visual attention range characteristic of SZ. By contrast, in least discriminative images, both groups primarily attend to the most prominent objects, resulting in minimal differences in the fixation density maps. This pattern can be attributed to the simple backgrounds and highly salient targets in line drawings and black-white images. Notably, highly discriminative images generally exhibit greater visual complexity, with richer scene details or multi-element layouts, whereas least discriminative images display simplified, low-complexity structures with limited visual variation.

**Figure 6:**
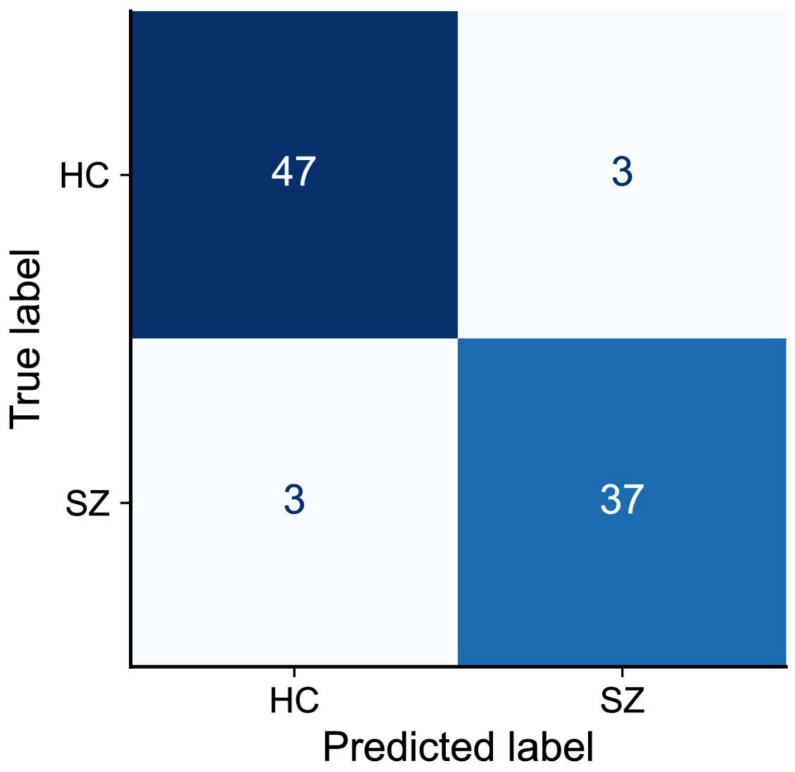
Confusion matrix of the best-performing classification system (SSKECA–XGBoost) based on the selected 25 semantic images. True and predicted labels correspond to HC and SZ. Numbers in each cell represent the number of subjects. Darker colors indicate higher counts, and lighter colors indicate lower counts.

As detailed in Section 2.8, a total of 25 images were selected across categories based on a classification accuracy threshold of 0.85. Model performance metrics evaluated on the selected images are summarized in Table 6. XGBoost achieved the highest accuracy (0.922), with a 95% CI of 0.800–0.941, indicating consistent performance and stability. The corresponding confusion matrix is shown in Figure 6, providing a subject-level visualization of correctly and incorrectly classified HC and SZ participants. Compared with the results reported in Table 4, a key finding is that the proposed framework maintains robust and consistent performance when processing eye-tracking data derived from semantically rich images, even under a substantially reduced sample size. Overall, most classifiers exhibit competitive and consistent performance across all evaluation metrics, including accuracy, precision, recall, F1-score, and AUC.

### 3.4. Misclassification Analyses Results

**Figure 7:**
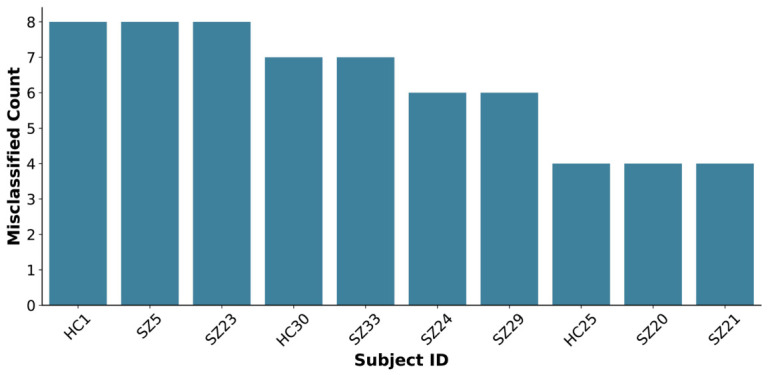
Top 10 subjects with the highest misclassification frequency. The number of misclassifications was calculated for each subject, and the 10 subjects with the highest counts are shown.

Misclassification analyses were performed within the subject-level five-fold stratified CV framework. For each subject, the ground-truth and predicted labels were recorded across all folds, and misclassification frequencies were calculated at the optimal feature dimension. Figure 7 shows the distribution of misclassification frequencies across all participants and highlights the top 10 most frequently misclassified subjects, with error counts ranging from 4 to 8. Notably, misclassifications were mainly concentrated in the SZ cohort, with only minimal occurrences in the HC group.

**Figure 8:**
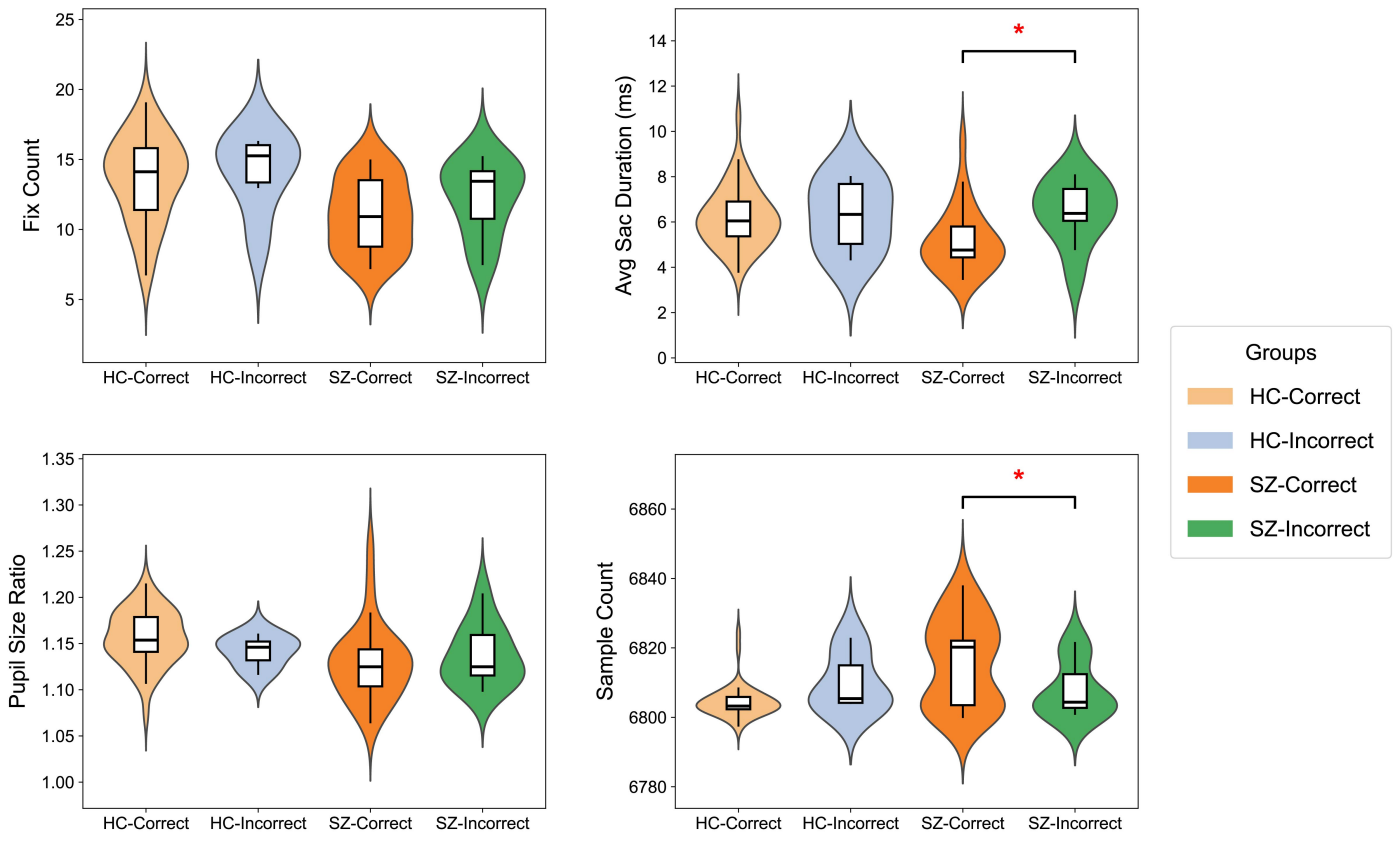
Distribution of representative eye-tracking features for correctly classified and misclassified subjects in the HC and SZ groups. Asterisks indicate significant differences between correctly classified and misclassified subjects within each group. * p<0.05.

To further characterize feature distribution patterns associated with classification errors, one representative feature was selected from each of the four feature categories, namely FF, SAF, PSF, and BF. As shown in Figure 8, HC subjects showed no significant differences between correctly classified and misclassified samples. In contrast, misclassified SZ patients exhibited significant alterations in multiple oculomotor indices within the SAF and BF feature sets (p<0.05), as shown in the two right panels of Figure 8. These findings suggest that model misclassifications in SZ may be associated with disease-related alterations in attentional and visual processing mechanisms, rather than random classification noise.

## 4. Discussion

This study systematically investigated eye movement biomarkers and visual attention patterns in SZ during a free-viewing task. By integrating comprehensive eye movement data with a semantic image-based SSKECA method, we developed an objective and quantifiable approach for differentiating SZ patients from HC at the individual level. The results demonstrated significant abnormalities in visual attention allocation and pupil dynamics among SZ patients. These alterations may reflect dysfunction in neural circuits implicated in SZ and provide objective indicators with discriminative potential. The proposed framework addresses the pressing need for measurable, mechanism-informed biomarkers in biological psychiatry [37] and provides a practical technical basis for the objective, quantifiable characterization of SZ-related eye movement abnormalities.

**Abnormal Eye Movement Metrics and Underlying Neural Mechanisms.** SZ patients exhibited widespread abnormalities in eye movement metrics, reflecting deficits in neural cognitive processing rather than merely peripheral oculomotor dysfunction. Statistical analyses revealed significant intergroup differences (p<0.05) across multiple feature categories, with the proportion of significant indicators within each category as follows: SAF (100%), FF (22.22%), PSF (28.57%), and BF (75%). These findings support the sensitivity of these eye movement biomarkers and are consistent with the spectrum of SZ-related eye movement abnormalities reported in large-sample studies [9]. Notably, saccade count emerged as a key discriminative feature and may serve as a behavioral proxy for dysfunction in the prefrontal–basal ganglia circuitry, which plays a critical role in executive control and is frequently implicated in SZ [38,39,40]. Key anomalies, including reduced average saccade velocity and amplitude, may reflect alterations in neural systems involving the dorsolateral prefrontal cortex (DLPFC) and the parietal attention network. These findings are consistent with the hypofrontality hypothesis [41] and converge with neuroimaging evidence demonstrating attenuated DLPFC activation in SZ [23,42,43], supporting the role of prefrontal dysfunction in the disorder. Additionally, BF metric abnormalities (e.g., prolonged duration and increased frequency) indicate disturbances in visual information processing. Several clinically relevant features also showed directional changes, including reduced fixation skewness, shorter valid viewing duration, and increased outside fixation counts, although only valid viewing duration reached statistical significance. Collectively, these multidimensional eye movement features provide convergent evidence of altered visual information processing in SZ and further support the sensitivity and neurocognitive relevance of the identified feature set. Notably, the present study further demonstrated that SZ subjects prone to misclassification displayed more severe alterations in certain eye movement metrics. This observation may reveal that such eye movement deficits represent core disease-related characteristics rather than random noise, thereby contributing to ambiguous classification boundaries.

**Pupillary Dynamics as Expanded Physiological Indicators for SZ.** By incorporating multidimensional pupil features into the eye movement analysis framework, this study expands the scope of physiological indicators relevant to SZ. The results showed that basic pupil size (e.g., average pupil size, minimum pupil size, and maximum pupil size) was similar between groups, yet SZ patients exhibited impaired pupil variability, characterized by lower pupil size ratio. Such alterations may reflect dysregulation in neural systems involving cortico-limbic circuits associated with arousal and cognitive control. The dynamic range of pupil size, which is influenced by the locus coeruleus–norepinephrine (LC-NE) system, has been reported to correlate with negative symptoms (e.g., blunted affect and avolition) and cognitive deficits in SZ [44,45]. These observations suggest potential disturbances in autonomic and arousal regulation during social cognitive processing. Although the gaze y-position at maximum pupil size exhibited no significant univariate group difference, it was identified as a critical feature in the SSKECA-derived feature importance analysis. This pattern may reflect altered arousal states linked to LC-NE pathway activity and prefrontal cortical function [46]. Collectively, these findings provide additional physiological insights into the visual and social cognitive impairments observed in SZ and highlight the potential relevance of pupil-related features for understanding the underlying neurocognitive mechanisms [47]. Importantly, these eye movement and pupillary abnormalities were not expressed uniformly across conditions but varied with the semantic and social characteristics of the visual stimuli.

**Visual Exploration Patterns Under Semantic Image Stimulation.** Through a systematic preference analysis of 20 semantic image categories, this study identified a distinctive pattern of visual exploration in SZ. Patients exhibited the most pronounced eye movement abnormalities when viewing images with high semantic complexity and strong social relevance (e.g., social interaction scenes and emotional faces), allowing the model to reach at least the preset accuracy threshold of 0.85. In contrast, low-complexity images (e.g., line drawings) provided insufficient information for effective differentiation, exhibiting limited discriminatory power within the model. These results suggest impairments in processing visual stimuli that demand semantic interpretation, contextual integration, and social-emotional reasoning, aligning with recent research on visual cognition in SZ [48]. Furthermore, this pattern may also be interpreted within the framework of predictive coding theories of SZ. Previous work suggests that disruptions in feedforward-feedback neural signaling may impair the generation of effective top-down predictions that guide visual exploration [49], thereby reducing the efficiency of processing complex and context-rich information. Overall, these findings suggest that visual processing abnormalities in SZ may vary across stimulus types rather than reflecting a uniform oculomotor deficit, providing further insight into the neurocognitive characteristics of the disorder [50,51]. Capturing these context-dependent and potentially subtle abnormalities from high-dimensional eye movement data requires robust feature optimization and integration methods, which is achieved through the SSKECA method developed in this study.

**SSKECA Method Advantages and Clinical Application.** The SSKECA method introduced in this study constitutes a clinically relevant, mechanism-driven feature optimization strategy designed to extract stable and neurobiologically interpretable biomarkers from high-dimensional and complex eye movement data. By integrating sparse representation and entropy contribution theory within a kernel framework, SSKECA can effectively reduce the influence of individual variability and noise features irrelevant to disease pathology while prioritizing the retention of informative eye movement indicators. These biomarkers may be associated with the function of core neural circuits, such as the prefrontal-parietal attention network and the LC-NE system, thereby achieving the dual goals of feature dimensionality reduction and optimization. In addition, this innovative feature transformation paradigm helps address the limitations of traditional statistical analysis methods. For example, although blink count and average fixation *x*-axis resolution initially showed no significant intergroup differences in univariate analysis (p>0.05), their importance scores exceeded 0.1 after SSKECA-based transformation and feature fusion, establishing them as meaningful auxiliary features that contribute to improved diagnostic performance. Compared with conventional dimensionality reduction techniques such as KECA, PCA, and KPCA [52], SSKECA demonstrates superior performance in extracting feature subsets with greater interpretability and neurophysiological relevance, thereby providing strong technical support for accurate SZ identification.

To further enhance diagnostic efficiency and accuracy, SSKECA was integrated with the AdaBoost algorithm, which optimizes the integration of weak classifiers through dynamic sample reweighting [53], a mechanism that is highly compatible with the enhanced feature representations produced by SSKECA. Together, these approaches enabled the development of a high-performance individualized assessment model by integrating a hybrid eye movement feature matrix (including fixation, saccade, pupil, and biological features) with SSKECA-based transformation and multiple classifiers. This SSKECA–AdaBoost model addressed the key limitation of current SZ diagnosis, which primarily depends on subjective interviews and behavioral observations, lacking standardized tools and therefore being susceptible to clinician experience and patient-reported biases [2]. The results demonstrated that the SSKECA–AdaBoost model achieved an accuracy of 0.933 ± 0.061, representing an improvement of approximately 4% compared with the model using the original feature matrix and outperforming conventional dimensionality-reduction baselines in classification accuracy. Notably, the XGBoost classifier achieved the optimal accuracy (0.922 ± 0.063) using only 25 semantic images, corresponding to a testing duration of approximately three minutes. The results suggest that the SSKECA combined with ML framework robustly extracts SZ-relevant eye movement biomarkers that remain stable when participants view semantically rich stimuli (e.g., social interaction and emotional images). These stimuli may activate the SZ-related cortico-striato-thalamo-cortical (CSTC) network [54], thereby supporting the model’s clinical relevance and diagnostic reliability. In addition, such efficiency is particularly important for clinical translation, as patients with SZ often struggle with prolonged assessments due to cognitive fatigue and attentional deficits. This finding is consistent with current calls for brief and well-tolerated diagnostic tools in psychosis research [55].

**Limitations and Future Directions.** The present study has several limitations. First, the sample was not stratified by disease stage, and medication effects were not controlled. Future large-scale longitudinal studies should include drug-naïve first-episode patients to track biomarker evolution and to assess their potential utility for early diagnosis and treatment monitoring. Second, no external validation dataset was included in the current study, as all data were collected under the same experimental setting. Therefore, the generalizability of the proposed model still needs to be further evaluated in independent cohorts. Third, the use of static images and the evaluation of SSKECA only in specific eye movement tasks may limit the ecological and clinical applicability of the present findings. Future studies should incorporate more naturalistic stimuli, such as dynamic video materials, and examine the robustness of SSKECA across additional paradigms. Finally, the current study relied solely on eye movement as a single modality. Future research should explore multimodal frameworks by integrating features such as facial and head movements to construct more robust identification models [56].

## 5. Conclusions

In this study, we developed an SSKECA paired-classifier framework for SZ identification, representing a novel hybrid architecture that integrates sparse representation, kernel methods, and entropy theory. When coupled with AdaBoost, the proposed approach achieved a classification accuracy of 0.933 ± 0.061, which compared favorably to conventional dimensionality-reduction techniques such as PCA, KPCA, and KECA. A distinctive characteristic of this framework is the construction of a composite feature matrix that systematically integrates fixation, saccade, pupil, and biological parameters. In addition, semantic analyses indicate that discriminative eye movement patterns are more pronounced for specific stimulus categories, allowing classification performance to be preserved with a substantially reduced set of stimuli. This “few-image” paradigm shortens data acquisition time and reduces operational burden in clinical settings, thereby improving practical feasibility. Collectively, these findings demonstrate that the SSKECA–ML framework provides a robust and efficient approach for extracting diagnostically relevant eye movement patterns in SZ, with potential for future clinical application.

## Figures and Tables

**Table 1:** Demographic and clinical characteristics of the SZ group and HC group.

	SZ	HC	$t/\chi^{2}$	*p*-Value
Number	40	50	–	–
Age (year)	34.80 ± 11.90	32.40 ± 5.82	2.18	<0.05 *
Gender (male/female)	28/12	29/21	0.91	0.34
Education (year)	11.90 ± 1.13	13.40 ± 1.39	5.65	<0.001 ***
PANSS scores	75.0 ± 10.8	–	–	–

Note: Variable values are presented as mean ± SD. PANSS: Positive and Negative Symptom Scale. * p<0.05, *** p<0.001.

**Table 2:** The details of hybrid feature set.

Feature Name	Definition	HC	SZ	*p*-Value	*t*-Value	Cohen’s *d*
**FF**						
Fix Count	Number of fixations	13.39 ± 2.86	11.20 ± 2.41	<0.001 ***	3.942	0.821
Avg Fix Duration (ms)	Average fixation duration	375.18 ± 172.80	372.82 ± 127.16	0.941	0.074	0.015
Max Fix Duration (ms)	Maximum fixation duration	795.31 ± 343.54	787.60 ± 245.97	0.902	0.124	0.025
Fix Skewness	Fixation skewness	163.69 ± 25.15	155.04 ± 27.09	0.124	1.554	0.332
Fix Duration (ms)	Total fixation duration	3789.46 ± 513.26	3493.68 ± 641.91	0.020 *	2.370	0.515
Outside Fixation Count	Fixations outside screen area	0.49 ± 1.50	0.58 ± 0.75	0.720	−0.360	−0.071
Avg Fix X Resolution (${/}^{{}^{\circ}}$)	Average fixation *x*-axis resolution	23.08 ± 0.12	23.10 ± 0.21	0.481	−0.709	−0.159
Avg Fix Y Resolution (${/}^{{}^{\circ}}$)	Average fixation *y*-axis resolution	22.68 ± 0.18	22.79 ± 0.39	0.110	−1.624	−0.371
Min Fix Duration (ms)	Minimum fixation duration	148.05 ± 88.72	137.51 ± 62.00	0.510	0.662	0.135
**SAF**						
Avg Sac Velocity	Average saccade velocity	144.72 ± 38.59	79.44 ± 31.54	<0.001 ***	8.830	1.832
Sac Amplitude (°)	Saccade amplitude	132.73 ± 75.80	63.72 ± 20.30	<0.001 ***	6.167	1.187
Sac Count	Number of saccades	13.83 ± 2.86	11.69 ± 2.37	<0.001 ***	3.891	0.809
Avg Sac Duration (ms)	Average saccade duration	6.27 ± 1.36	5.45 ± 1.32	0.005 **	2.881	0.609
**PSF**						
DR of Pupil Size	Dynamic range of pupil size	0.36 ± 0.06	0.30 ± 0.09	0.002 **	3.267	0.717
Pupil Size Ratio	Pupil size ratio	1.15 ± 0.03	1.13 ± 0.04	0.011 *	2.608	0.574
Min Pupil Size	Minimum pupil size	1439.83 ± 527.37	1669.00 ± 785.94	0.119	−1.581	−0.350
Avg Pupil Size	Average pupil size	1823.20 ± 675.95	2007.52 ± 906.47	0.288	−1.070	−0.234
Max Pupil Size	Maximum pupil size	2096.02 ± 769.00	2263.21 ± 1007.77	0.389	−0.867	−0.189
Y to Max Pupil Size	Gaze y position at max pupil size	325.66 ± 28.34	333.79 ± 47.74	0.345	−0.952	−0.213
X to Max Pupil Size	Gaze x position at max pupil size	381.52 ± 39.28	383.04 ± 51.49	0.878	−0.155	−0.034
**BF**						
Duration (ms)	Total gaze duration	6804.59 ± 5.37	6811.59 ± 10.42	<0.001 ***	−3.858	−0.874
Sample Count	Number of valid eye movement sample	6803.99 ± 5.47	6810.59 ± 10.42	<0.001 ***	−3.625	−0.820
Valid Viewing Duration (ms)	Valid gaze duration	4708.35 ± 201.55	4605.75 ± 220.41	0.025 *	2.279	0.488
Blink Count	Number of blinks	2.53 ± 1.27	3.32 ± 2.42	0.067	−1.869	−0.423

Note: Variable values are presented as mean ± SD. The reported *t*-values and *p*-values were derived from Welch’s two-sample *t*-test. * p<0.05, ** p<0.01, *** p<0.001. Bold abbreviations denote feature subsets: **FF** = Fixation features, **SAF** = Saccade features, **PSF** = Pupil size features, **BF** = Biological features.

**Table 3:** Comparative effectiveness of different dimensionality reduction algorithms.

Algorithm	Best Classifier	Dim	Accuracy (95% CI)	Precision (95% CI)	Recall (95% CI)	F1-Score (95% CI)	AUC (95% CI)
Original	AdaBoost	24	0.900 ± 0.082 (0.822–0.944)	0.904 ± 0.093 (0.831–0.967)	0.875 ± 0.177 (0.700–0.950)	0.880 ± 0.107 (0.784–0.948)	0.963 ± 0.046 (0.908–0.990)
**SSKECA**	**AdaBoost**	**21**	**0.933 ± 0.061** **(0.871–0.967)**	**0.927 ± 0.068** **(0.870–0.978)**	**0.925 ± 0.112** **(0.800–0.975)**	**0.923 ± 0.074** **(0.840–0.963)**	**0.960 ± 0.054** **(0.900–0.988)**
KECA	AdaBoost	15	0.922 ± 0.063 (0.867–0.967)	0.924 ± 0.070 (0.870–0.978)	0.900 ± 0.105 (0.800–0.950)	0.910 ± 0.076 (0.843–0.962)	0.930 ± 0.046 (0.900–0.972)
KPCA	AdaBoost	17	0.911 ± 0.050 (0.845–0.933)	0.886 ± 0.079 (0.819–0.933)	0.925 ± 0.069 (0.875–0.950)	0.903 ± 0.052 (0.848–0.938)	0.948 ± 0.050 (0.905–0.980)
PCA	AdaBoost	14	0.911 ± 0.101 (0.822–0.978)	0.933 ± 0.099 (0.800–0.978)	0.875 ± 0.217 (0.551–0.975)	0.886 ± 0.142 (0.733–0.976)	0.958 ± 0.055 (0.905–0.988)

Note: Values represent mean ± SD, with the 95% BCa bootstrap CIs reported in parentheses below. The “Original” row refers to the full feature set without dimensionality reduction. Bold font indicates the proposed algorithm.

**Table 4:** Robustness analysis of the SSKECA algorithm across different classifiers.

Classifier	Dim	Accuracy (95% CI)	Precision (95% CI)	Recall (95% CI)	F1-Score (95% CI)	AUC (95% CI)
AdaBoost	21	0.933 ± 0.061 (0.871–0.967)	0.927 ± 0.068 (0.870–0.978)	0.925 ± 0.112 (0.800–0.975)	0.923 ± 0.074 (0.840–0.963)	0.960 ± 0.054 (0.900–0.988)
MLP	22	0.922 ± 0.063 (0.867–0.956)	0.927 ± 0.068 (0.870–0.978)	0.900 ± 0.137 (0.750–0.950)	0.908 ± 0.079 (0.840–0.960)	0.970 ± 0.047 (0.910–0.993)
SVM	14	0.911 ± 0.050 (0.856–0.933)	0.902 ± 0.056 (0.870–0.956)	0.900 ± 0.105 (0.800–0.950)	0.898 ± 0.062 (0.828–0.928)	0.943 ± 0.048 (0.905–0.975)
XGBoost	14	0.911 ± 0.063 (0.867–0.956)	0.949 ± 0.071 (0.870–0.978)	0.850 ± 0.137 (0.750–0.900)	0.891 ± 0.079 (0.834–0.948)	0.948 ± 0.053 (0.898–0.980)
RF	21	0.900 ± 0.120 (0.789–0.976)	0.938 ± 0.091 (0.837–0.978)	0.825 ± 0.244 (0.575–0.925)	0.865 ± 0.169 (0.708–0.970)	0.953 ± 0.062 (0.894–0.995)
LightGBM	13	0.889 ± 0.104 (0.788–0.944)	0.938 ± 0.091 (0.836–0.978)	0.800 ± 0.209 (0.600–0.900)	0.854 ± 0.147 (0.703–0.931)	0.965 ± 0.058 (0.887–0.995)
CNN	20	0.889 ± 0.096 (0.739–0.933)	0.960 ± 0.089 (0.840–1.000)	0.775 ± 0.163 (0.541–0.850)	0.855 ± 0.138 (0.703–0.933)	0.950 ± 0.072 (0.858–0.985)
KNN	13	0.878 ± 0.127 (0.756–0.944)	0.928 ± 0.110 (0.800–0.978)	0.775 ± 0.256 (0.500–0.900)	0.831 ± 0.195 (0.600–0.931)	0.963 ± 0.064 (0.877–0.993)

Note: Values represent mean ± SD, with 95% BCa bootstrap CIs reported in parentheses below.

**Table 5:** The experimental results of different methods on the SZ identification task.

Methods	Accuracy	Precision	Recall	F1	AUC
GPI–LSTM [34]	0.711 ± 0.127	0.729 ± 0.189	0.700 ± 0.112	0.692 ± 0.066	0.770 ± 0.180
GPI–GRU [34]	0.767 ± 0.138	0.803 ± 0.211	0.650 ± 0.240	0.702 ± 0.185	0.808 ± 0.132
ABG–LSTM [35]	0.756 ± 0.084	0.721 ± 0.079	0.750 ± 0.234	0.719 ± 0.120	0.788 ± 0.109
**SSKECA–AdaBoost**	** 0.933 ± 0.061 **	** 0.927 ± 0.068 **	** 0.925 ± 0.112 **	** 0.923 ± 0.074 **	** 0.960 ± 0.054 **

Note: Performance is presented as mean ± SD. The best-performing method is highlighted in bold.

**Table 6:** Model performance attained by SSKECA based on selected images.

Classifier	Dim	Accuracy (95% CI)	Precision (95% CI)	Recall (95% CI)	F1-Score (95% CI)	AUC (95% CI)
XGBoost	24	0.922 ± 0.063 (0.800–0.941)	0.927 ± 0.068 (0.870–0.978)	0.900 ± 0.137 (0.600–0.950)	0.908 ± 0.079 (0.720–0.938)	0.960 ± 0.054 (0.900–0.993)
MLP	24	0.911 ± 0.063 (0.789–0.944)	0.909 ± 0.089 (0.843–0.949)	0.900 ± 0.137 (0.650–0.925)	0.897 ± 0.077 (0.731–0.931)	0.965 ± 0.039 (0.904–0.990)
AdaBoost	20	0.900 ± 0.107 (0.767–0.933)	0.938 ± 0.091 (0.778–0.971)	0.825 ± 0.209 (0.650–0.925)	0.869 ± 0.151 (0.734–0.932)	0.925 ± 0.059 (0.912–0.990)
LightGBM	21	0.900 ± 0.082 (0.767–0.944)	0.919 ± 0.076 (0.840–0.978)	0.850 ± 0.163 (0.500–0.900)	0.878 ± 0.107 (0.679–0.938)	0.953 ± 0.057 (0.910–0.988)
Random Forest	24	0.889 ± 0.088 (0.767–0.956)	0.944 ± 0.079 (0.836–0.960)	0.800 ± 0.190 (0.500–0.900)	0.856 ± 0.118 (0.691–0.965)	0.960 ± 0.054 (0.897–0.995)
SVM	20	0.889 ± 0.068 (0.800–0.933)	0.872 ± 0.023 (0.800–0.933)	0.875 ± 0.153 (0.800–0.950)	0.869 ± 0.093 (0.788–0.928)	0.928 ± 0.053 (0.905–0.972)
CNN	24	0.889 ± 0.111 (0.775–0.922)	0.909 ± 0.089 (0.780–0.931)	0.825 ± 0.209 (0.632–0.925)	0.858 ± 0.154 (0.729–0.915)	0.935 ± 0.070 (0.886–0.985)
KNN	21	0.856 ± 0.115 (0.767–0.944)	0.898 ± 0.095 (0.824–0.972)	0.750 ± 0.198 (0.550–0.900)	0.813 ± 0.157 (0.684–0.933)	0.951 ± 0.051 (0.872–0.982)

Note: Performance is presented as mean ± SD, with the 95% BCa bootstrap CIs reported in parentheses below.

## Data Availability

The data that support the findings of this research are available on reasonable request from the corresponding author. The data are not publicly available due to privacy or ethical restrictions.

## Supplementary Materials

The following supporting information can be downloaded at: https://www.mdpi.com/article/10.3390/jemr19030051/s1, Table S1. Total number of images per category; Table S2. Hyperparameter configurations for all classifiers; Table S3. Comprehensive performance metrics for all algorithm-classifier combinations; Figure S1. A density scatter plot depicting the association between sample and subject-level feature importance.

## References

[1] Miley K., Bronstein M.V., Ma S., Lee H., Green M.F., Ventura J., Hooker C.I., Nahum M., Vinogradov S. (2024). Trajectories and Predictors of Response to Social Cognition Training in People with Schizophrenia: A Proof-of-Concept Machine Learning Study. Schizophr. Res..

[2] Pan B., Li X., Weng J., Xu X., Yu P., Zhao Y., Yu D., Zhang X., Tang X. (2025). Identifying Periphery Biomarkers of First-Episode Drug-Naïve Patients with Schizophrenia Using Machine-Learning-Based Strategies. Prog.-Neuro-Psychopharmacol. Biol. Psychiatry.

[3] Kay S.R., Fiszbein A., Opler L.A. (1987). The Positive and Negative Syndrome Scale (PANSS) for Schizophrenia. Schizophr. Bull..

[4] Sheehan D.V., Lecrubier Y., Sheehan K.H., Amorim P., Janavs J., Weiller E., Hergueta T., Baker R., Dunbar G.C. (1998). The Mini-International Neuropsychiatric Interview (MINI): The Development and Validation of a Structured Diagnostic Psychiatric Interview for DSM-IV and ICD-10. J. Clin. Psychiatry.

[5] Oh S., Nairuz T., Park S.J., Lee J.H. (2026). Simultaneous Analysis of Microsaccades and Pupil Size Variations in Age-Related Cognitive Impairment Using Eye-Tracking Technology. J. Eye Mov. Res..

[6] Okada K.I., Miura K., Fujimoto M., Morita K., Yoshida M., Yamamori H., Yasuda Y., Iwase M., Inagaki M., Shinozaki T. (2021). Impaired Inhibition of Return during Free-Viewing Behaviour in Patients with Schizophrenia. Sci. Rep..

[7] Okazaki K., Miura K., Matsumoto J., Hasegawa N., Fujimoto M., Yamamori H., Yasuda Y., Makinodan M., Hashimoto R. (2023). Discrimination in the Clinical Diagnosis between Patients with Schizophrenia and Healthy Controls Using Eye Movement and Cognitive Functions. Psychiatry Clin. Neurosci..

[8] Sprenger A., Trillenberg P., Nagel M., Sweeney J.A., Lencer R. (2013). Enhanced Top-down Control during Pursuit Eye Tracking in Schizophrenia. Eur. Arch. Psychiatry Clin. Neurosci..

[9] Dong Z., Chen H., Zhu R.S., Jia G., Liang Y. (2025). The Diagnostic Role of Exploratory Eye Movement in Schizophrenia: A Systematic Review and Meta-Analysis. BMC Psychiatry.

[10] Qiu L., Yan H., Zhu R., Yan J., Yuan H., Han Y., Yue W., Tian L., Zhang D. (2018). Correlations between Exploratory Eye Movement, Hallucination, and Cortical Gray Matter Volume in People with Schizophrenia. BMC Psychiatry.

[11] Gu Y., Li Y., Xu L., Zhang T., Cui H., Wei Y., Xia M., Su W., Tang Y., Tang X. (2025). Predictive Role of Fixation Stability for Clinical Stages and Conversion in Schizophrenia and Its Correlation with Cognitive Function. Schizophr. Bull..

[12] Zhang D., Xu L., Xie Y., Tang X., Hu Y., Liu X., Wu G., Qian Z., Tang Y., Liu Z. (2023). Eye Movement Indices as Predictors of Conversion to Psychosis in Individuals at Clinical High Risk. Eur. Arch. Psychiatry Clin. Neurosci..

[13] Li S., Liu Z., Zhang D., Song Y., Xu L., Zhang T., Wang J. (2026). Schizophrenia Recognition via Eye Movement Features in Video Paradigm. Eur. Arch. Psychiatry Clin. Neurosci..

[14] Lyu H., St Clair D., Wu R., Benson P.J., Guo W., Wang G., Liu Y., Hu S., Zhao J. (2023). Eye Movement Abnormalities Can Distinguish First-Episode Schizophrenia, Chronic Schizophrenia, and Prodromal Patients From Healthy Controls. Schizophr. Bull. Open.

[15] Huang L., Wei W., Liu Z., Zhang T., Wang J., Xu L., Chen W., Le Meur O. (2020). Effective Schizophrenia Recognition Using Discriminative Eye Movement Features and Model-Metric Based Features. Pattern Recognit. Lett..

[16] Iwauchi K., Tanaka H., Okazaki K., Matsuda Y., Uratani M., Morimoto T., Nakamura S. (2023). Eye-Movement Analysis on Facial Expression for Identifying Children and Adults with Neurodevelopmental Disorders. Front. Digit. Health.

[17] Chen Z., Ou Y., Ding Y., Wang Y., Li H., Liu F., Li P., Lv D., Liu Y., Lang B. (2025). Abnormal Eye Movement, Brain Regional Homogeneity in Schizophrenia and Clinical High-Risk Individuals and Their Associated Gene Expression Profiles. Schizophrenia.

[18] Song Y., Liu Z., Li G., Xie J., Wu Q., Zeng D., Xu L., Zhang T., Wang J. (2025). EMS: A Large-Scale Eye Movement Dataset, Benchmark, and New Model for Schizophrenia Recognition. IEEE Trans. Neural Netw. Learn. Syst..

[19] Jenssen R. (2009). Kernel Entropy Component Analysis. IEEE Trans. Pattern Anal. Mach. Intell..

[20] Zhang D., Liu X., Xu L., Li Y., Xu Y., Xia M., Qian Z., Tang Y., Liu Z., Chen T. (2022). Effective Differentiation between Depressed Patients and Controls Using Discriminative Eye Movement Features. J. Affect. Disord..

[21] Parisot K., Zozor S., Guérin-Dugué A., Phlypo R., Chauvin A. (2021). Micro-Pursuit: A Class of Fixational Eye Movements Correlating with Smooth, Predictable, Small-Scale Target Trajectories. J. Vis..

[22] Manor B.R., Gordon E. (2003). Defining the Temporal Threshold for Ocular Fixation in Free-Viewing Visuocognitive Tasks. J. Neurosci. Methods.

[23] Morita K., Miura K., Kasai K., Hashimoto R. (2020). Eye Movement Characteristics in Schizophrenia: A Recent Update with Clinical Implications. Neuropsychopharmacol. Rep..

[24] Skaramagkas V., Giannakakis G., Ktistakis E., Manousos D., Karatzanis I., Tachos N.S., Tripoliti E., Marias K., Fotiadis D.I., Tsiknakis M. (2021). Review of Eye Tracking Metrics Involved in Emotional and Cognitive Processes. IEEE Rev. Biomed. Eng..

[25] Liu X., Li Y., Xu L., Zhang T., Cui H., Wei Y., Xia M., Su W., Tang Y., Tang X. (2024). Spatial and Temporal Abnormalities of Spontaneous Fixational Saccades and Their Correlates with Positive and Cognitive Symptoms in Schizophrenia. Schizophr. Bull..

[26] Wu H., Li F., Chu W., Li H., Ji Y., Li Y., Niu Y., Wang H., Chen Y., Shi G. (2025). A Novel RSVP-Based System Using EEG and Eye-Movement for Classification and Localization. Biomed. Signal Process. Control.

[27] Tsui H.K.H., Liao Y., Hsiao J.H.W., Suen Y.N., Yan E.W.C., Poon L.T., Siu M.W., Hui C.L.M., Chang W.C., Lee E.H.M. (2025). Eye Movement Abnormalities During the Gaze Perception Task in Individuals with Clinical High Risk for Psychosis: A Discriminant Analysis with Hidden Markov Models. Schizophr. Bull..

[28] Nielsen J.R.W., Dietz M., Jefsen O.H. (2025). Blink Rates in Patients with Schizophrenia Compared to Healthy Controls: A Meta-Analysis. Schizophr. Res..

[29] Han H., Li D., Liu W., Zhang H., Wang J. (2024). High Dimensional Mislabeled Learning. Neurocomputing.

[30] Hu T., Li Q., Liu S., Calhoun V.D., van Wingen G., Yu S. (2026). BrainIB++: Leveraging Graph Neural Networks and Information Bottleneck for Functional Brain Biomarkers in Schizophrenia. Biomed. Signal Process. Control.

[31] Barone P. (2014). Kernel Density Estimation via Diffusion and the Complex Exponentials Approximation Problem. Q. Appl. Math..

[32] Bareis N., Wang Y., Olfson M., Gerhard T., Dixon L., Stroup T.S. (2025). Machine Learning for Novel Phenotyping in Schizophrenia. Schizophr. Res..

[33] De Miras J.R., Ibáñez-Molina A.J., Soriano M.F., Iglesias-Parro S. (2023). Schizophrenia Classification Using Machine Learning on Resting State EEG Signal. Biomed. Signal Process. Control.

[34] Zhou W., Yang M., Tang J., Wang J., Hu B. (2024). Gaze Patterns in Children with Autism Spectrum Disorder to Emotional Faces: Scanpath and Similarity. IEEE Trans. Neural Syst. Rehabil. Eng..

[35] Li J., Chen Z., Zhong Y., Lam H.K., Han J., Ouyang G., Li X., Liu H. (2022). Appearance-Based Gaze Estimation for ASD Diagnosis. IEEE Trans. Cybern..

[36] Kim J., Park I.H. (2024). Identifying an Early Neuropathological Mechanism in Schizophrenia with Brain Organoids. Biol. Psychiatry.

[37] Jang K., Li L., Le T.H., Setiani A., Rami F.Z., Kim H., Chung Y.C. (2025). Acoustic Biomarkers for Schizophrenia Spectrum Disorders and Their Associations with Symptoms and Cognitive Functioning. Prog.-Neuro-Psychopharmacol. Biol. Psychiatry.

[38] Lencer R., Trillenberg P. (2008). Neurophysiology and Neuroanatomy of Smooth Pursuit in Humans. Brain Cogn..

[39] Woodward N.D., Karbasforoushan H., Heckers S. (2012). Thalamocortical Dysconnectivity in Schizophrenia. Am. J. Psychiatry.

[40] Khalil M., Hollander P., Raucher-Chéné D., Lepage M., Lavigne K.M. (2022). Structural Brain Correlates of Cognitive Function in Schizophrenia: A Meta-Analysis. Neurosci. Biobehav. Rev..

[41] Weinberger D.R. (1987). Implications of Normal Brain Development for the Pathogenesis of Schizophrenia. Arch. Gen. Psychiatry.

[42] Voineskos A.N., Hawco C., Neufeld N.H., Turner J.A., Ameis S.H., Anticevic A., Buchanan R.W., Cadenhead K., Dazzan P., Dickie E.W. (2024). Functional Magnetic Resonance Imaging in Schizophrenia: Current Evidence, Methodological Advances, Limitations and Future Directions. World Psychiatry.

[43] Wang Y.J., Wen Y., Zheng L., Chen J., Lin Z., Pan Y. (2025). A Computational and Multi-Brain Signature for Aberrant Social Coordination in Schizophrenia. Prog.-Neuro-Psychopharmacol. Biol. Psychiatry.

[44] Mäki-Marttunen V., Andreassen O.A., Espeseth T. (2020). The Role of Norepinephrine in the Pathophysiology of Schizophrenia. Neurosci. Biobehav. Rev..

[45] Suttkus S., Schumann A., De La Cruz F., Bär K.J. (2021). Working Memory in Schizophrenia: The Role of the Locus Coeruleus and Its Relation to Functional Brain Networks. Brain Behav..

[46] Joshi S., Gold J.I. (2020). Pupil Size as a Window on Neural Substrates of Cognition. Trends Cogn. Sci..

[47] Bismark A.W., Mikhael T., Mitchell K., Holden J., Granholm E. (2024). Pupillary Responses as a Biomarker of Cognitive Effort and the Impact of Task Difficulty on Reward Processing in Schizophrenia. Schizophr. Res..

[48] Qela B., Damiani S., De Santis S., Groppi F., Pichiecchio A., Asteggiano C., Brondino N., Monteleone A.M., Grassi L., Politi P. (2025). Predictive Coding in Neuropsychiatric Disorders: A Systematic Transdiagnostic Review. Neurosci. Biobehav. Rev..

[49] Sterzer P., Adams R.A., Fletcher P., Frith C., Lawrie S.M., Muckli L., Petrovic P., Uhlhaas P., Voss M., Corlett P.R. (2018). The Predictive Coding Account of Psychosis. Biol. Psychiatry.

[50] Kutlikova H.H., Čavojská N., Ivančík V., Straková A., Januška J., Pečeňák J., Heretik A., Hajdúk M. (2025). Visual Processing of Social and Non-Social Stimuli in Schizophrenia: Investigation of the Links to Positive and Negative Symptoms. Cogn. Neuropsychiatry.

[51] Yang E., Tadin D., Glasser D.M., Hong S.W., Blake R., Park S. (2013). Visual Context Processing in Schizophrenia. Clin. Psychol. Sci..

[52] Sadegh-Zadeh S.A., Sadeghzadeh N., Soleimani O., Ghidary S.S., Movahedi S., Mousavi S.Y. (2024). Comparative Analysis of Dimensionality Reduction Techniques for EEG-Based Emotional State Classification. Am. J. Neurodegener. Dis..

[53] Latreche I., Slatnia S., Kazar O., Harous S., Khelili M.A. (2024). Identification and Diagnosis of Schizophrenia Based on Multichannel EEG and CNN Deep Learning Model. Schizophr. Res..

[54] Liddle P.F., Sami M.B. (2025). The Mechanisms of Persisting Disability in Schizophrenia: Imprecise Predictive Coding via Corticostriatothalamic-Cortical Loop Dysfunction. Biol. Psychiatry.

[55] Weng T., Zheng Y., Xie Y., Qin W., Guo L. (2024). Diagnosing Schizophrenia Using Deep Learning: Novel Interpretation Approaches and Multi-Site Validation. Brain Res..

[56] Khan W., Topham L., Alsmadi H., Al Kafri A., Kolivand H. (2024). Deep Face Profiler (DeFaP): Towards Explicit, Non-Restrained, Non-Invasive, Facial and Gaze Comprehension. Expert Syst. Appl..

