# Supplementary material for: Eye Movement Patterns as Robust Biomarkers for Schizophrenia Identification Using a Novel Data Transformation Approach
Source: J Eye Mov Res. 2026 May 11;19(3):51. doi: 10.3390/jemr19030051 (PMC13214958; doi:10.3390/jemr19030051)
Supplement: Supplementary file 1 [file jemr-19-00051-s001.zip › jemr-4249030-supplementary.pdf]

Supplementary materials

Table S1. Total number of images per category

Class	Category	Total No.
1	Action	10
2	Affection	6
3	Art	6
4	Black-white	2
5	Cartoon	4
6	Fractal	3
7	Indoor scenes	6
8	Inverted images	5
9	Image patches	4
10	Line drawings	2
11	Low resolution	7
12	Noisy images	7
13	Objects	5
14	Outdoor man-made	4
15	Outdoor natural scenes	7
16	Patterns	5
17	Randomly captured images	2
18	Satellite images	3
19	Sketches	3
20	Social scenes	9

Table S2 Hyperparameter configurations for all classifiers

Classifier	Key Hyperparameters
XGBoost	n_estimators=300, random_state=42, eval_metric=logloss
LightGBM	n_estimators=300, random_state=42
Random Forest	n_estimators=300, random_state=42
SVM	RBF kernel, C=1, class_weight=balanced, probability=True
AdaBoost	n_estimators=300, random_state=42
MLP	hidden_layer_sizes=(100,), max_iter=300
KNN	n_neighbors=7
1D-CNN	Conv1D(32,3), Conv1D(16,3), Dense(32), Dropout(0.3), Adam, batch=8, epochs=50

Table S3. Comprehensive performance metrics for all algorithm-classifier combinations

Algorithm	Classifier	Dimension	Accuracy	Precision	Recall	F1-Score	AUC
Original	AdaBoost	24	0.900 ± 0.082	0.904 ± 0.093	0.875 ± 0.177	0.880 ± 0.107	0.963 ± 0.046
Original	KNN	24	0.756 ± 0.115	0.821 ± 0.208	0.550 ± 0.259	0.637 ± 0.249	0.870 ± 0.082
Original	LightGBM	24	0.889±0.056	0.924±0.070	0.825±0.143	0.864±0.077	0.968±0.041
Original	SVM	24	0.733 ± 0.107	0.833 ± 0.204	0.475 ± 0.224	0.589 ± 0.233	0.898 ± 0.102
Original	RF	24	0.900 ± 0.091	0.928 ± 0.110	0.850 ± 0.163	0.879 ± 0.112	0.958 ± 0.050
Original	MLP	24	0.856 ± 0.084	0.812 ± 0.118	0.900 ± 0.137	0.847 ± 0.094	0.930 ± 0.072
Original	XGBoost	24	0.900±0.072	0.924±0.070	0.850±0.163	0.877±0.096	0.968±0.036
Original	CNN	24	0.867±0.108	0.834±0.152	0.900±0.163	0.857±0.125	0.953±0.051
SSKECA	AdaBoost	21	0.933±0.061	0.927±0.068	0.925±0.112	0.923±0.074	0.960±0.054
SSKECA	KNN	13	0.878±0.127	0.928±0.110	0.775±0.256	0.831±0.195	0.963±0.064
SSKECA	LightGBM	13	0.889±0.104	0.938±0.091	0.800±0.209	0.854±0.147	0.965±0.058
SSKECA	MLP	22	0.922±0.063	0.927±0.068	0.900±0.137	0.908±0.079	0.970±0.047
SSKECA	RandomForest	21	0.900±0.120	0.938±0.091	0.825±0.244	0.865±0.169	0.953±0.062
SSKECA	SVM	14	0.911±0.050	0.902±0.056	0.900±0.105	0.898±0.062	0.943±0.048
SSKECA	XGBoost	14	0.911±0.063	0.949±0.071	0.850±0.137	0.891±0.079	0.948±0.053
SSKECA	CNN	20	0.889±0.096	0.960±0.089	0.775±0.163	0.855±0.138	0.950±0.072
KECA	AdaBoost	15	0.922±0.063	0.924±0.070	0.900±0.105	0.910±0.076	0.930±0.046
KECA	KNN	16	0.878±0.091	0.922±0.075	0.800±0.227	0.839±0.137	0.950±0.054
KECA	LightGBM	22	0.911±0.075	0.927±0.068	0.875±0.177	0.890±0.100	0.970±0.047
KECA	MLP	16	0.911±0.075	0.900±0.061	0.900±0.163	0.894±0.101	0.968±0.053
KECA	RandomForest	14	0.889±0.088	0.919±0.076	0.825±0.190	0.860±0.118	0.945±0.059
KECA	SVM	19	0.900±0.072	0.866±0.096	0.925±0.112	0.891±0.082	0.960±0.050
KECA	XGBoost	13	0.889±0.056	0.927±0.068	0.825±0.168	0.862±0.079	0.940±0.060
KECA	CNN	19	0.878±0.091	0.891±0.072	0.825±0.209	0.846±0.134	0.958±0.061
KPCA	AdaBoost	17	0.911±0.050	0.886±0.079	0.925±0.069	0.903±0.052	0.948±0.050
KPCA	KNN	6	0.878±0.099	0.944±0.079	0.775±0.224	0.836±0.143	0.940±0.063

Algorithm	Classifier	Dimension	Accuracy	Precision	Recall	F1-Score	AUC
KPCA	LightGBM	10	0.878±0.120	0.938±0.091	0.775±0.256	0.831±0.177	0.948±0.061
KPCA	MLP	16	0.889±0.068	0.897±0.062	0.850±0.163	0.866±0.093	0.965±0.038
KPCA	RandomForest	11	0.900±0.091	0.944±0.079	0.825±0.190	0.872±0.123	0.960±0.061
KPCA	SVM	18	0.889±0.068	0.866±0.096	0.900±0.137	0.876±0.079	0.958±0.047
KPCA	XGBoost	19	0.889±0.088	0.919±0.076	0.825±0.190	0.860±0.118	0.968±0.041
KPCA	CNN	20	0.911±0.084	0.922±0.075	0.875±0.177	0.891±0.111	0.960±0.051
PCA	AdaBoost	14	0.911±0.101	0.933±0.099	0.875±0.217	0.886±0.142	0.958±0.055
PCA	KNN	9	0.756±0.115	0.821±0.208	0.550±0.259	0.637±0.249	0.870±0.085
PCA	LightGBM	13	0.889±0.088	0.888±0.114	0.875±0.177	0.870±0.108	0.935±0.072
PCA	MLP	19	0.889±0.104	0.927±0.104	0.825±0.209	0.858±0.143	0.958±0.057
PCA	RandomForest	15	0.889±0.104	0.906±0.103	0.850±0.224	0.860±0.143	0.955±0.067
PCA	SVM	13	0.856±0.108	0.858±0.147	0.825±0.143	0.835±0.123	0.933±0.056
PCA	XGBoost	11	0.878±0.107	0.881±0.125	0.850±0.205	0.853±0.135	0.943±0.063
PCA	CNN	24	0.878±0.120	0.938±0.091	0.775±0.256	0.831±0.177	0.950±0.066

Note: Performance is presented as mean ± SD

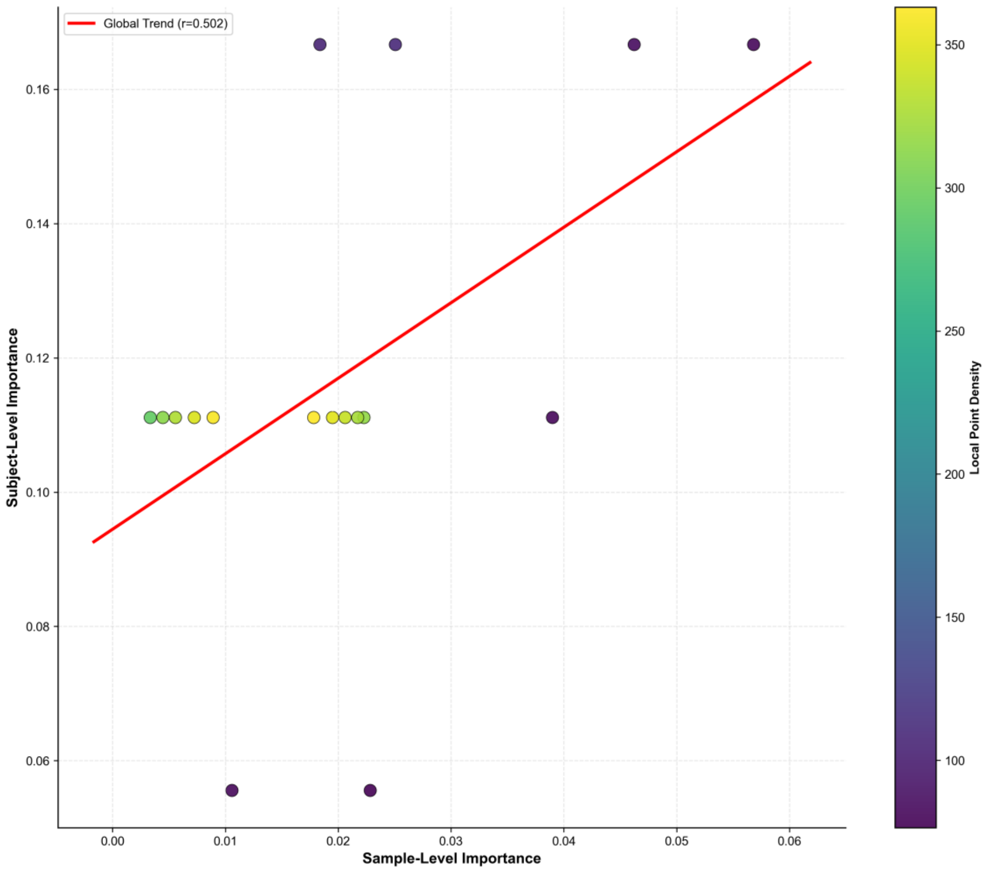

Fig. S1 A density scatter plot depicting the association between sample and subject-level feature importance.
